# Differentiation of pancreatic neuroendocrine carcinoma from pancreatic ductal adenocarcinoma using magnetic resonance imaging: The value of contrast-enhanced and diffusion weighted imaging

**DOI:** 10.18632/oncotarget.17309

**Published:** 2017-04-21

**Authors:** Chuangen Guo, Xiao Chen, Zhongqiu Wang, Wenbo Xiao, Qidong Wang, Ke Sun, Xiaoling Zhuge

**Affiliations:** ^1^ Department of Radiology, The First Affiliated Hospital, College of Medicine Zhejiang University, Hangzhou 310003, China; ^2^ Department of Radiology, The Affiliated Hospital of Nanjing University of Chinese Medicine, Nanjing 2100029, China; ^3^ Division of Nephrology, Zhongshan Hospital Fudan University, Shanghai 200032, China; ^4^ Department of Pathology, The First Affiliated Hospital, College of Medicine Zhejiang University, Hangzhou 310003, China; ^5^ Department of Laboratory Medicine, The First Affiliated Hospital, College of Medicine Zhejiang University, Hangzhou 310003, China

**Keywords:** pancreatic ductal adenocarcinoma, pancreatic neuroendocrine carcinoma, magnetic resonance imaging, diffusion-weighted imaging

## Abstract

Pancreatic neuroendocrine carcinoma (PNEC) is often misdiagnosed as pancreatic ductal adenocarcinoma (PDAC). This retrospective study differentiated PNEC from PDAC using magnetic resonance imaging (MRI), including contrast-enhanced (CE) and diffusion-weighted imaging (DWI). Clinical data and MRI findings, including the T1/T2 signal, tumor boundary, size, enhancement degree, and apparent diffusion coefficient (ADC), were compared between 37 PDACs and 13 PNECs. Boundaries were more poorly defined in PDAC than PNEC (97.3% vs. 61.5%, p<0.01). Hyper-/isointensity was more common in PNEC than PDAC at the arterial (38.5% vs. 0.0), portal (46.2% vs. 2.7%) and delayed phases (46.2% vs. 5.4%) (all p<0.01). Lymph node metastasis (97.3% vs. 61.5%, p<0.01) and local invasion/distant metastasis (86.5% vs. 46.2%, p<0.01) were more common in PDAC than PNEC. Enhancement degree via CE-MRI was higher in PNEC than PDAC at the arterial and portal phases (p<0.01). PNEC ADC values were lower than those of normal pancreatic parenchyma (p<0.01) and PDAC (p<0.01). Arterial and portal phase signal intensity ratios and ADC values showed the largest areas under the receiver operating characteristic curve and good sensitivities (92.1%–97.2%) and specificities (76.9%–92.3%) for differentiating PNEC from PDAC. Thus the enhancement degree at the arterial and portal phases and the ADC values may be useful for differentiating PNEC from PDAC using MRI.

## INTRODUCTION

Pancreatic ductal adenocarcinoma (PDAC) is the most common malignant tumor of the pancreas. It is highly aggressive and rapidly fatal, with a five-year survival rate <5% [1]. While resection can be curative, resection rates remain low at 10–15% [2] due to local invasion or distant metastases. The PDAC characteristic vascularization pattern can be visualized using computed tomography (CT) or magnetic resonance imaging (MRI) [3, 4], and PDACs are often hypovascularized as compared to adjacent normal tissue [5].

Pancreatic neuroendocrine neoplasms (PNENs) account for 1–2% of pancreatic tumor cases [6]. The WHO 2010 classification [7] separates PNENs into grade 1 (G1), grade 2 (G2), and neuroendocrine carcinoma (NEC, G3) based on histological differentiation, including mitoses and Ki-67 proliferation index. Pancreatic neuroendocrine carcinomas (PNECs) are very rare, accounting for only 2–3% of PNENs [8, 9]. PNENs are hypervascular lesions with marked enhancement via CT or MRI [10–13]. Jang, *et al*. [13] showed differences in tumor margin, enhancement pattern, bile duct dilatation, pancreatic duct dilatation, and pancreatic atrophy between PNENs and PDAC. Several studies also showed that contrast-enhanced ultrasonography (3D US and harmonic endoscopic US) is useful for differential diagnosis of PDAC and PNEN [14, 15]. Kim, *et al*. [16] observed differences in transfer coefficient (K(trans)), rate constant (K(ep)), and initial area under the concentration curve over 60 sec (iAUC) between PDAC and PNEN using dynamic contrast-enhanced MRI. However, recent studies indicated that G1 and G2 PNEN and PNEC enhancement patterns differ [11, 12, 17]. Most PNECs exhibit arterial and portal phase hypoenhancement, indicating that PNECs and PDACs have similar enhancement degrees. Kimura, *et al*. [18] reported a PNEC case, misdiagnosed as PDAC, that exhibited low vascularity on enhanced CT. Lewis, *et al*. [8] also demonstrated that PNEC MRIs resemble those of PDACs, including T1 and T2 signals, and hypoenhancement. Therefore, we speculate that qualitative imaging is not effective for differentiating PNEC from PDAC.

PDAC and PNEC treatment strategies and prognoses differ. For PNEC, surgery is indicated if curative resection is possible, even in those cases with limited metastases, for example to liver [19, 20]. In addition, targeted therapy with sunitinib or everolimus [21] and somatostatin analogues (octrecotide) [22], or radionuclide-labeled somatostatin (DOTATATE) [23] may be valuable for PNEC, along with cytotoxic chemotherapy (e.g., cisplatin with etoposide). PNEC are generally less aggressive and have better outcomes than PDAC. The PNEC five-year survival rate is approximately 27.7% [24], which is higher than that of PDAC (<5%). Pretreatment differentiation of PNEC from PDAC is important in determining therapeutic strategies. To the best of our knowledge, no study has explored differences in imaging features between PDAC and PNEC. MRI, particularly in diffusion-weight imaging (DWI), has been used to differentiate pancreatitis and pancreatic cancer [25–28], and MRI has a similar or better performance in PDAC evaluation [29, 30]. The present study assessed the value of MRI, including DWI and dynamic contrast-enhanced imaging, for differentiating PNEC and PDAC.

## RESULTS

### Patient and tumor characteristics

**Figure 1:**
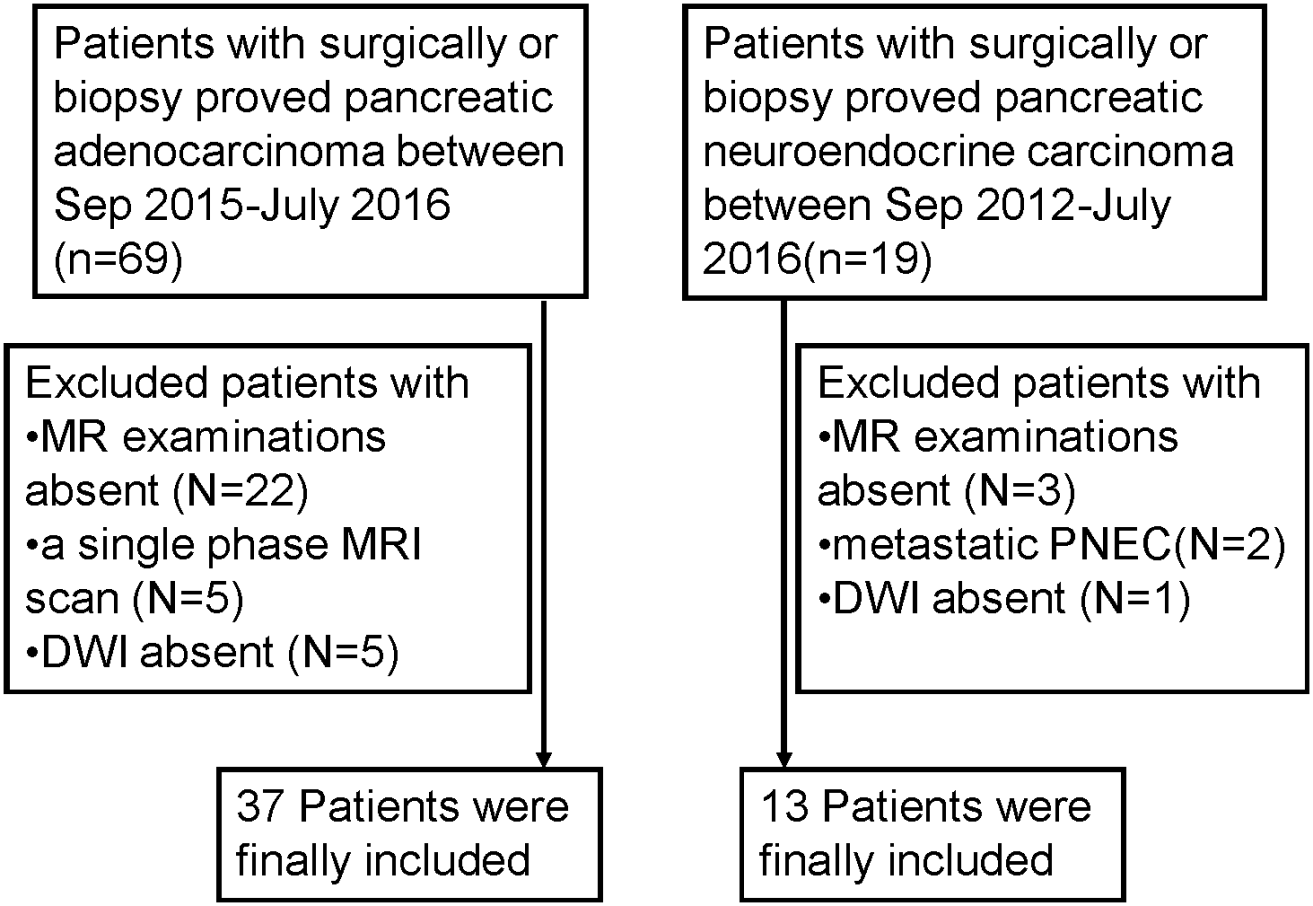
Flow diagram of the study patients with pancreatic ductal adenocarcinoma (PDAC) and pancreatic neuroendocrine carcinoma (PNEC)

Thirty-seven PDAC and 13 PNEC patients were analyzed (Figure 1, Table 1) in this retrospective study. Thirty-one PDAC patients underwent surgery and six underwent biopsy. Twelve PNEC patients underwent surgery and one underwent biopsy. We compared demographic data and clinical symptoms between PDAC and PNEC patients. No differences were found for age, gender, or clinical symptoms between those two groups. However, yellow urine or icterus was more common in PDAC compared with PNEC patients (27.0% vs. 7.7%, p>0.05). PNEC tended to occur in men compared with PDAC (76.9% vs. 62.2%, p>0.05). Carbohydrate antigen 19-9 (CA19-9) and CA125 levels were higher in PDAC than in PENC patients. Abnormal CA19-9 level was more common in PDAC than PNEC (81.8% vs 30.8%, p<0.05). In our series, 89.2% of PDAC patients were correctly diagnosed via MRI, while eight (61.5%) PNEC patients were misdiagnosed as PDAC via MRI.

**Table 1:** Clinical data of patients

Variables	PDAC(n=37)	PNEC(n=13)	P
Age (years)	61.2±8.5(43-79)	53.9±13.1(32-71)	0.06
Gender			>0.05
Male	23(62.2%)	10(76.9%)	
Female	14(37.8%)	3(23.1%)	
Clinical symptoms			
Abdominal Pain	19(51.4%)	10(76.9%)	>0.05
Confusion of consciousness or dizziness	0	1(7.7%)	>0.05
Diarrhea or Abdominal bloating	8(21.6%)	2(15.4%)	>0.05
Yellow urine or icterus	10(27.0%)	1(7.7%)	>0.05
Marasmus	4(10.8%)	0	>0.05
Others	2(5.4%)	1(7.7%)	>0.05
Asymptomatic	5(13.5%)	3(23.1%)	>0.05
Surgery	31(84.6%)	12(92.3%)	>0.05
Biopsy	6(15.4%)	1(7.7%)	>0.05
CA19-9 (U/ml)*	211.4(5-12000)	12.5(2.9-638)	<0.001
>37	30(81.8%)	4(30.8%)	<0.001
CA125(U/ml)*	21.9(6.6-188.5)	8.9(4.7-43.4)	<0.05
CEA(ng/ml)*	3.9(0.8-55.7)	2.3(0.9-159)	>0.05
Imaging diagnosis			<0.05
Pancreatic cancer	33(89.2%)	8(61.5%)	
PNEC	0	1(7.7%)	
Pancreatic cystadenoma or cystadenocarcinoma	2(5.4%)	0	
Others	2(5.4%)	4(30.7%)	

*Data are shown as median.

CA19-9: Carbohydrate antigen 19-9; CA125: Carbohydrate antigen 125; CEA: carcino-embryonic antigen; PNEC: pancreatic neuroendocrine carcinoma

### MRI findings: qualitative analysis

**Figure 2:**
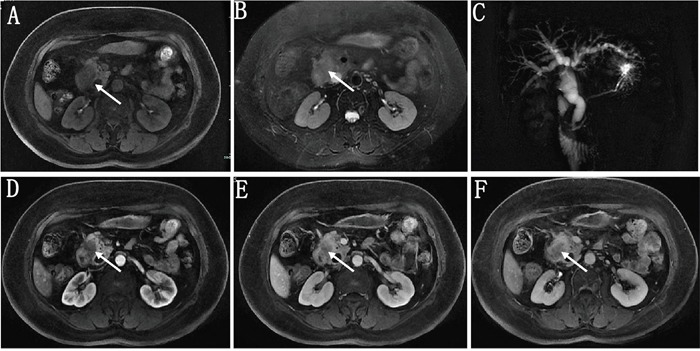
A 57 year old female patient with pathologically proven pancreatic ductal adenocarcinoma On fat-suppressed LAVA T1- **(A)** and T2- **(B)** weighted imaging, the tumor showed hypointensity and hyperintensity with an ill-defined boundary, respectively. MRI cholangiopancreatography (MRCP) showed the common bile duct and intra-, extrahepatic bile ducts were markedly dilated **(C)**. Gadolinium enhanced images in arterial **(D)**, portal **(E)** and delayed phase **(F)** both showed the tumor were hypointensity compared with the pancreatic parenchyma.

**Figure 3:**
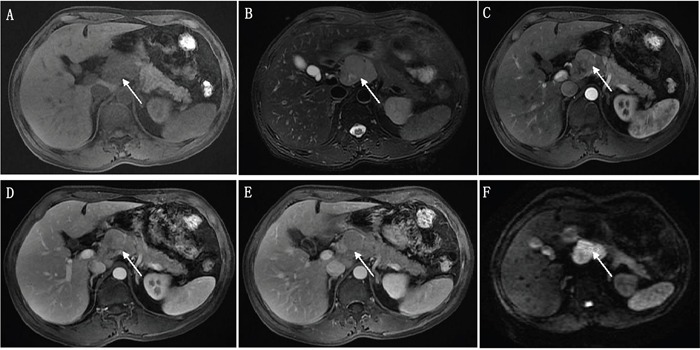
A 54 year old male patient with pathologically proven pancreatic neuroendocrine carcinoma The tumor showed hypointensity and hyperintensity with relatively well-defined boundary on fat-suppressed LAVA T1- **(A)** and T2- **(B)** weighted imaging, respectively. The tumor showed hypo- to isointensity at arterial phase **(C)**, isointensity at portal **(D)** and delayed phases **(E)** compared with the pancreatic parenchyma in contrast-enhanced images. Diffusion-weighted images showed the tumor was marked hyperintensity **(F)**.

Qualitative MRI data were summarized in Table 2. PDAC occurred more commonly in pancreatic head-neck compared with PNEC (70.3% vs. 46.2%), but the difference was not significant. A well defined boundary was more prevalent in PNEC compared with PDAC (38.5% vs. 2.7%, p<0.01). Differences in T1 signal intensity and DWI signal were not significant between PDAC and PNEC. However, isointensity in T2 weighted images was more common in PNEC compared with PDAC (23.1% vs. 2.7%, p=0.05). Moreover, hyper- and isointensity were more common in PNEC than PDAC at arterial phase (38.5% vs. 0.0, p<0.01), portal phase (46.2% vs. 2.7%, p<0.01), and delayed phase (46.2% vs. 5.4%, p< 0.01). Lymph node metastases and local invasion/distant metastases were more common in PDAC than PNEC (97.3% vs. 61.5%, 86.5 vs. 46.2%, respectively, p<0.01). Intra/extrahepatic bile duct dilatations were also more common in PDAC than in PNEC (54.1% vs. 23.1%), but this difference was not significant. Representative unenhanced T1- and T2-weighted PDAC and PNEC images, and gadolinium-enhanced images are shown in Figures 2 and 3. PDAC showed hypointensity in T1 weighted images, hyperintensity in T2 weighted images, and marked hypointensity compared with normal pancreas in contrast-enhanced images. Figure 2C showed bile duct dilatation in PDAC. PNEC also showed hypointensity in T1 weighted images (Figure 3A), hyperintensity in T2 weighted images (Figure 3B), and isointensity in contrast-enhanced T1 weighted images (Figure 3C–3E). Additionally, PNEC boundaries (Figure 3) were relatively well defined compared with those of PDAC (Figure 2).

**Table 2:** The summary of MRI findings

MR findings	PDAC(n=37)	PNEC(n=13)	p
Location			>0.05
Pancreatic Head-neck	26(70.3%)	6(46.2%)	
Pancreatic Body-tail	11(29.7%)	7(53.9%)	
Boundary			<0.01
Well-circumscribed	1(2.7%)	5((38.5%)	
Ill-defined	36(97.3%)	8(61.5%)	
MRI signal of tumor			
TIWI			>0.05
Isointense	4(10.8%)	3(23.1%)	
Hypointense	29(78.4%)	10(76.9%)	
Iso-/Hypo intensity	4(10.8%)	0	
T2WI			0.05
Isointense	1(2.7%)	3(23.1%)	
Hyperintense	36(97.3%)	10(76.9%)	
DWI			0.15
Isointense	2(5.4%)	0	
Moderate Hyperintense	8(21.6%)	1(7.7%)	
Marked Hyperintense	27(73.0%)	12(92.3%)	
Enhancement degree			
Arterial phases			<0.01
Hyper- intense	0	1(7.7%)	
Iso-intense	0	4(30.8%)	
Hypointense*	37(100%)	8(61.5%)	
Portal phases			<0.01
Hyper-intense	0	1(7.7%)	
Iso-intense	1(2.7%)	5(38.5%)	
Hypo-intense*	36(97.3%)	7(53.8%)	
Delayed phases			
Hyperintense	0	1(7.7%)	<0.01
Iso-intense	2(5.4%)	5(38.5%)	
Hypointense*	35(94.6)	7(53.8%)	
Pancreatic duct dilatation	26(70.5%)	8(61.5%)	>0.05
Intra-, extrahepatic bile duct dilatation	20(54.1%)	3(23.1%)	>0.05
Pancreatic atrophy	8(21.6%)	3(23.1%)	>0.05
Lymphnodes invasion	36(97.3%)	8(61.5%)	<0.01
Local invasion or Metastases	32(86.5%)	6(46.2%)	<0.01
Size (cm)	3.3±1.5(1.2-7.1)	5.2±4.4(2.2-19)	0.03

*compared with hyper- and isointensity

DWI: diffusion weighted imaging

### MRI findings: quantitative analyses

**Figure 4:**
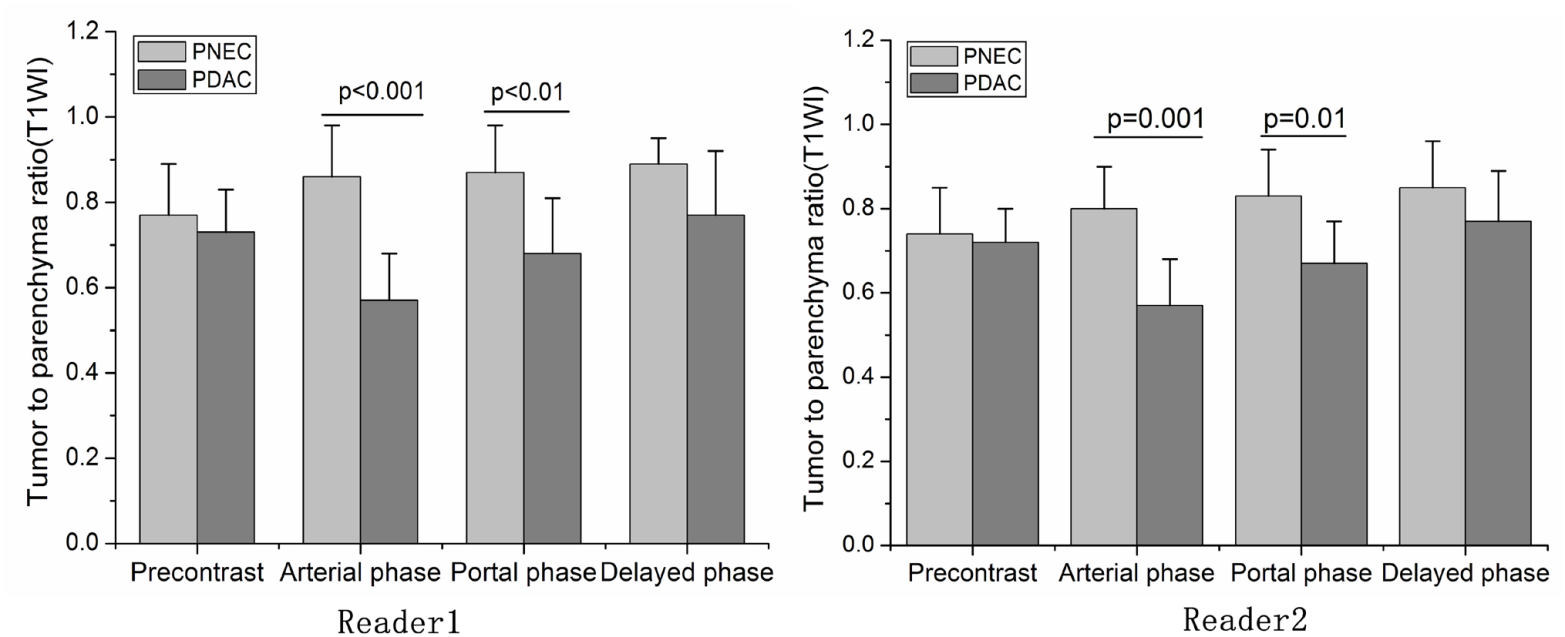
The signal intensity ratio compared with parenchyma in pancreatic ductal adenocarcinoma (PDAC) and pancreatic neuroendocrine carcinoma (PNEC) calculated by two readers Signal intensity ratios in PDAC were lower than PNEC at arterial and portal phases. * p≤ 0.01, compared with PDAC. The bias between the two readers were -1.2%(−9.6%, 7.2%), -2.2% (−14.0%, 9.5%) and 1.4% (−6.8%, 9.5%) for signal intensity ratio at arterial, portal and delayed phases, respectively.

We quantitatively analyzed tumor sizes, signal intensities in T1 weighted images, and apparent diffusion coefficient (ADC) values. Mean PNEC tumor size was greater than that of PDAC (5.2 cm vs. 3.3 cm, p=0.03) (Table 2). Figure 4 shows the signal intensity ratio in unenhanced (pre-contrast image) and contrast-enhanced T1 weighted images measured by two readers. PDAC signal intensity ratios were lower than those of PNEC at arterial and portal phases (p≤0.01) (Figure 4).

**Figure 5:**
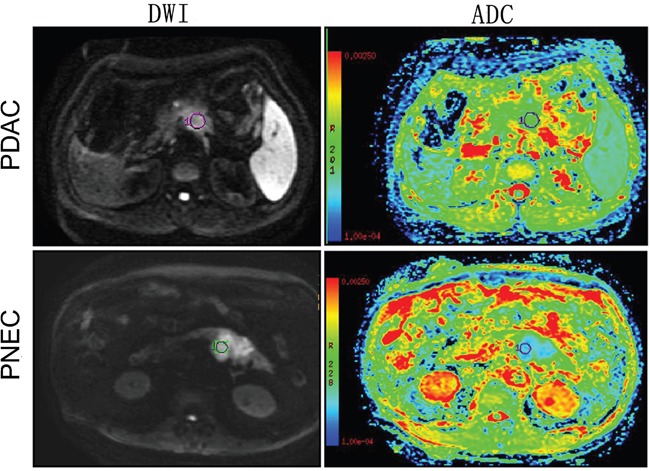
Diffusion-weighted images and ADC maps in pancreatic ductal adenocarcinoma (PDAC) and pancreatic neuroendocrine carcinoma (PNEC) PNEC showed higher DWI signal and lower ADC value than PDAC.

**Figure 6:**
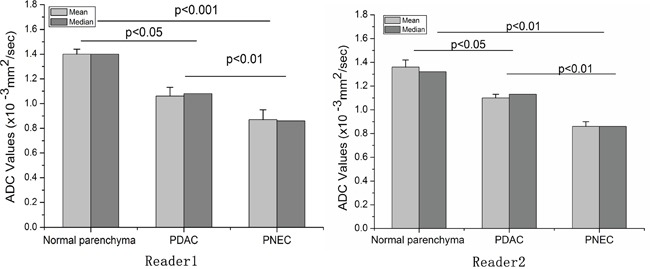
Apparent diffusion coefficients (ADC) values in pancreatic ductal adenocarcinoma (PDAC) and pancreatic neuroendocrine carcinoma (PNEC) measured by two readers The ADC values of PDAC and PNEC were both lower than the normal parenchyma. In addition, the ADC value of PNEC was also lower than PDAC. The bias between the two readers was -0.3%(−10.7%, 10%) for ADC value.

Representative DWI and ADC value maps for PDAC and PNEC are shown in Figure 5. PDAC DWI signal intensity was lower than that of PNEC. Mean ADC values for pancreatic parenchyma, PDAC, and PNEC were 1.38, 1.04, and 0.87×10^−3^ mm^2^/s (pooled data), respectively (Figure 6). In both readers, mean PDAC and PNEC ADC values were lower than in normal pancreas parenchyma (p<0.05 or 0.01). PNEC ADC values were also lower compared with those of PDAC (p< 0.01).

In addition, we also evaluated the measurement agreement between the readers. The correlations between the two readers were 0.87-0.93 for ADC values and signal intensity ratio. The bias between the two readers were -0.3%(−10.7%, 10%) for ADC value, -1.2%(−9.6%, 7.2%) for signal intensity ratio at arterial phase, -2.2%(−14.0%, 9.5%) at portal phase and 1.4% (−6.8%, 9.5%) at delayed phase.

### Imaging feature diagnostic performances

**Figure 7:**
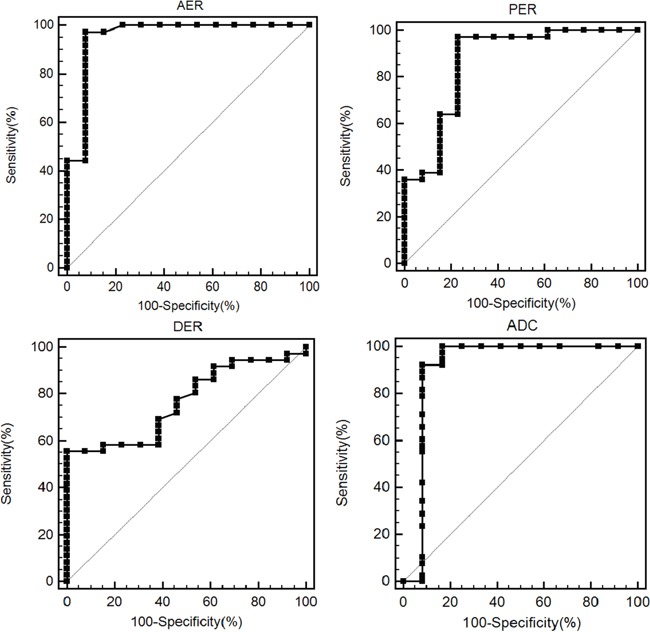
ROC curve of the signal intensity ratio at arterial (AER), portal (PER) and delayed phases (DER), and mean apparent diffusion coefficients (ADC) value for differentiating neuroendocrine carcinoma (PNEC) from pancreatic ductal adenocarcinoma (PDAC) The data from two readers were pooled together. The area under the curve is 0.954, 0.865, 0.769 and 0.910, respectively.

The sensitivity and specificity of the different imaging features for PNEC identification (vs. PDAC) ranged from 52.6%–97.2% and 38.5%–100%, respectively (Table 3). The area under the curve (AUC) ranged from 0.667–0.954 (Table 3). Signal intensity ratio at arterial and portal phases, and ADC value had the largest AUC, indicating that these features can potentially differentiate PNEC from PDAC (Figure 7). Cutoff values were 0.768 for signal intensity ratio at arterial phase with 97.2% sensitivity and 92.3% specificity, 0.823 for signal intensity ratio at portal phase with 97.2% sensitivity and 76.9% specificity, and 1.0×10^−3^ mm^2^/s for ADC values with 92.1% sensitivity and 91.7% specificity.

**Table 3:** Diagnostic performances of clinical and imaging features

Variables	AUC	Sensitivity(95%CI)(%)	Specificity(95%CI)(%)
AER	0.954	97.2(85.5-99.9)	92.3(64.0-99.8)
PER	0.865	97.2(85.5-99.9)	76.9(46.2-95.0)
DER	0.769	55.6(38.1-72.1)	100(75.3-100)
ADC	0.910	92.1(78.6-98.3)	91.7(61.5-99.8)
Sizes	0.694	70.1(52.5-83.9)	65.3(31.6-86.7)
Bile duct dilatation	0.680	52.6(35.8-69.0)	83.3(51.6-97.9)
Invasion or Metastases	0.769	87.2(72.6-95.7)	66.7(34.9-90.1)
Boundary	0.667	94.9(82.7-99.4)	38.5(13.9-68.4)
CA19-9	0.840	78.1(60.0-90.7)	78.6(54.4-93.9)

The data from two readers were pooled together.

AER: signal intensity ratio at arterial phase; PER: signal intensity ratio at portal phase; DER: signal intensity ratio at delayed phase; ADC: apparent diffusion coefficients; AUC: area under curve; CA19-9: Carbohydrate antigen 19-9; CI: confidential interval

## DISCUSSION

PNEC can mimic PDAC in CT or MR images due in part to a characteristic hypoenhanced pattern [8, 18]. It is valuable to accurately diagnose patient tumor type (PNEC vs. PDAC) before surgery, because treatment strategies and prognoses differ between the two types. The present study compared PNEC MR imaging features with those of PDAC. We found that ill-defined tumor boundaries, hypointensity in contrast-enhanced images, lymph node metastases, and local invasion/distant metastases were more common in PDAC than PNEC. Quantitative data further indicated that signal intensity ratios at arterial and portal phases, and ADC values have potential for differentiating those two tumors.

PDAC is a fast-progressing malignancy with surrounding tissue invasion and metastases to distant organs. Tumors usually exhibit ill-defined margins [13]. Lymph nodes metastases and local invasion or distant metastases are also common in PDAC [31]. In our series, 97% of PDAC cases had ill-defined margins and 97% exhibited lymph node metastases, which was consistent with previous studies. PNECs also frequently exhibited ill-defined boundaries (61%), and often metastasized to lymph nodes (61%) or invaded the surrounding tissues. However, these features were still more common in PDAC than PNEC. To some degree, these qualitative features may be useful for differentiating PNEC from PDAC.

Contrast-enhanced CT and MRI are helpful for differential diagnosis of benign and malignant lesions based on characteristic vascularization patterns [13, 32, 33]. Several studies showed differences between pancreatic carcinoma and mass-forming focal pancreatitis using contrast-enhanced approaches [31, 33]. PDACs are frequently hypovascular, and enhancement degree is lower than the surrounding pancreatic parenchyma, in particular at arterial and portal phases. However, most PNECs also showed hypoenhancement at arterial and portal phases. Thus, it is challenging to discriminate PNEC from PDAC using regularly qualitative features in contrast-enhanced images. We speculated that quantitative analysis of imaging features would provide more useful diagnostic information. Our data showed that PNEC enhancement degree is higher than that of PDAC at arterial and portal phases. The signal intensity ratios at arterial and portal phases showed good sensitivity and specificity in differentiating PNEC from PDAC. Our results confirmed that quantitative assessment provides reliable information in differentiating PNEC and PDAC.

DWI has also been widely used to differentiate benign and malignant pancreatic lesions [27, 33], and may be applicable for grading PNENs [12, 34] and differentiating PNENs and PDAC [35, 36]. Lee, *et al*. and Wang, *et al*. found no differences in ADC values between PNENs and PDAC [35, 36]. However, G1 and G2 PNETs, which may have higher ADC values than PNECs [12, 35], were included in these studies. Therefore, we speculate that it is important to consider PNEN grade when evaluating ADC values. As PNECs frequently exhibited high mitotic ability (>20 mitoses per 10 high powered fields (HPFs)) and proliferation (Ki-67 index >20%) in our study, PNECs should therefore exhibit high tissue cellularity and restricted water mobility. Our data supported this speculation. PNEC ADC values were lower than those of PDAC. Moreover, ADC showed very good sensitivity and specificity in differentiating PNEC from PDAC.

Our study had several limitations. First, retrospective studies may exhibit selection and verification bias. Some PNEC patients were excluded due to lack of MRI examinations, and the PNEC study population could not reflect the entire PNEC spectrum. Second, only CE-MR and routine DWI were studied, and some novel approaches, such as intravoxel incoherent motion (IVIM) DWI and texture analysis, would provide more information [37, 38]. Finally, larger number of *b* values would be more sensible rather to use only two *b* values [37].

In conclusion, we compared PDAC and PNEC MR imaging features, and found that ill-defined margins, lymph node metastases, and local invasion are more common in PDAC. In addition, PNEC enhancement degree at arterial and portal phases are higher than those of PNEC. PNEC usually exhibits lower ADC values than PDAC. Enhancement degree at arterial and portal phases and ADC value may be useful for differentiating PNEC from PDAC.

## MATERIALS AND METHODS

### Patient selection

This study included patients treated for PDAC and PNEC at our institution between September 2015 and July 2016, and September 2012 and July 2016, respectively. A total of 69 patients with surgically or biopsy diagnosed PDAC were identified. Twenty-two patients were excluded due to lack of MRI examination. Ten patients were excluded because of only a single-phase scan (n=5) or lack of DWI (n=5). We identified 19 patients with surgically or biopsy diagnosed PNEC. Six were excluded due to metastatic NEC (n=2), or lack of MRI examination (n=3) or DWI (n=1). In total, 37 PDAC and 13 PNEC patients were included in this study (Figure 1). The pathological diagnosis of PNEC was based on the WHO 2010 classification for NENs: NEC G3, >20 mitoses per 10 HPF, Ki-67 index >20%. In addition, demographic and clinical data were retrieved from medical records. This retrospective study was approved by our institutional review board with waiver of patient formal consent.

### MRI examinations

All MR scans were performed on a 3.0 superconducting system (Signa HDx 3.0-T, GE Medical Systems, USA) using eight-channel phased-array torso coils. Patients fasted for 8 h prior to MR examination. The protocol included 3D T1-weighted fat-suppressed liver acquisition with volume acceleration-extended volume (LAVA-XV, GE) [TR/TE: 3.1/1.5 ms; imaging duration, 1–2 min]; fast spin-echo T2-weighted fat-suppressed sequence (TE/TR: 4000–8000/80–90 ms; imaging duration, 2–3min) with 3–5 mm slice thickness, 1–2 mm interslice gap, 384×256 matrix and 300–400 mm field-of-view, and the axial DW sequence using the respiratory-triggered single shot echo-planar sequence [TR/TE: 6000–8000/60–70 ms; imaging duration, 2–3 min] with *b* values of 0 and 1000 s/mm^2^ before contrast administration. Based on the DWI signal at two different *b* values, tissue ADC values were obtained. MRI cholangiopancreatography (MRCP) was performed using heavily T2-weighted fast acquisition spin echo sequence (TR/TE: 2500–6000 /500–800 ms, imaging duration, 2.5 min). Gadopentetate dimeglumine (Magnevist, Bayer HealthCare Pharmaceuticals, Berlin, Germany) was injected at a dose of 0.1 mmol/kg of body weight (2.5 ml/s) following by a 20-ml saline flush, and then axial and coronal T1 images were obtained at 25–35 s (arterial phases), 60–70 s (portal phases) and 200–240 s (delayed phase).

### MR imaging analysis

All MR images were retrospectively reviewed by two abdominal radiologists with more than eight years of experience in abdominal MRI examination on a picture archiving communication system (PACS) workstation. The radiologists were blinded to the final histopathological results and MR diagnosis. The following imaging information was reviewed: tumor position (head-neck or body-tail), tumor margin [well-defined: smooth or lobulating margin with few spiculations or infiltrations (<20%); ill-defined: the perimeter of the tumor showed spiculation or infiltration (>20%)] (11), size, presence of cystic components (solid, cystic components <25%; or mixed cystic-solid), T1 and T2 signal, signal on DWI, enhancement degree at arterial, portal, and delayed phases (hypo-, iso-, or hyperintense compared with normal pancreas). The presence of intrahepatic/extrahepatic bile duct dilatation, pancreatic duct dilatation, pancreatic parenchymal atrophy, lymph node metastases, and local invasion/distant metastases were also reviewed. Pancreatic duct dilation was defined as the main pancreatic duct diameter ≥4 mm. Intrahepatic and extrahepatic bile duct dilatation were confirmed if the duct diameter was >5 mm and >8 mm, respectively. Areas that were hypointense on precontrast T1-weighted images, markedly hyperintense on T2-weighted images, and with no enhancement were identified as cystic components.

Quantitative analyses were performed using ADC values and tumor signal intensities on unenhanced and enhanced T1 weighted images by two abdominal radiologists. The signal intensity ratio of tumor to pancreas [signal intensity ratio=signal intensities of tumors/normal pancreatic parenchyma] was calculated. Cystic components were avoided during signal intensity analysis. On DWI and T1 weighted images, regions of interest (ROI) were centered on the solid tumor portions while avoiding necrotic or cystic components, and the most peripheral portions that might result in partial volume effects of adjacent extra-lesional tissues. Tumor and pancreatic parenchyma ADC values were measured. For the normal pancreas, signal intensities and ADC values were noted at a similar ROI avoiding the main duct. ADC values and signal intensities were measured at least three times by each radiologist. The means and the agreement between the two readers were analyzed.

### Pathology analysis

Tumor tissue specimens were fixed with 10% formalin, embedded in paraffin, and sliced for hematoxylin-eosin (H&E) staining. PDAC was typically characterized by moderately to poorly differentiated glandular structures. PNEC was diagnosed based on the 2010 WHO classification for neuroendocrine neoplasms by counting the number of mitoses per 10 HPFs and assessing Ki-67 proliferation index (percentage of positive cells in areas of highest nuclear labeling). NEC G3 was regarded as >20 mitoses per 10 HPF, Ki-67 index >20%.

### Statistical analysis

Data were analyzed using SPSS 16.0 (SPSS Inc, Chicago, IL). Quantitative data were presented as means (standard deviation) or median, and were analyzed via independent samples t test, Mann-Whitney U test, or one-way analysis of variance (ANOVA). Categorical variables were represented as the number of cases (percentage) and were analyzed using Chi-square or Fisher's exact tests when necessary. The data from the two readers were pooled together for diagnostic performance analysis. The diagnostic values, sensitivities, and specificities of ADC and signal intensity ratio for differentiating PDAC from PENC were assessed by the receiver operating characteristic (ROC) curve, and inter-readers agreement were determined by Bland-Altman plot using Medcalc software (Mariakerke, Belgium). P<0.05 was considered statistically significant.
